# Supplementary material for: Impact of fat mass and distribution on lipid turnover in human adipose tissue
Source: Nat Commun. 2017 May 23;8:15253. doi: 10.1038/ncomms15253 (PMC5457499; doi:10.1038/ncomms15253)
Supplement: Supplementary Information — Supplementary Figures, Supplementary Tables. [file ncomms15253-s1.pdf]

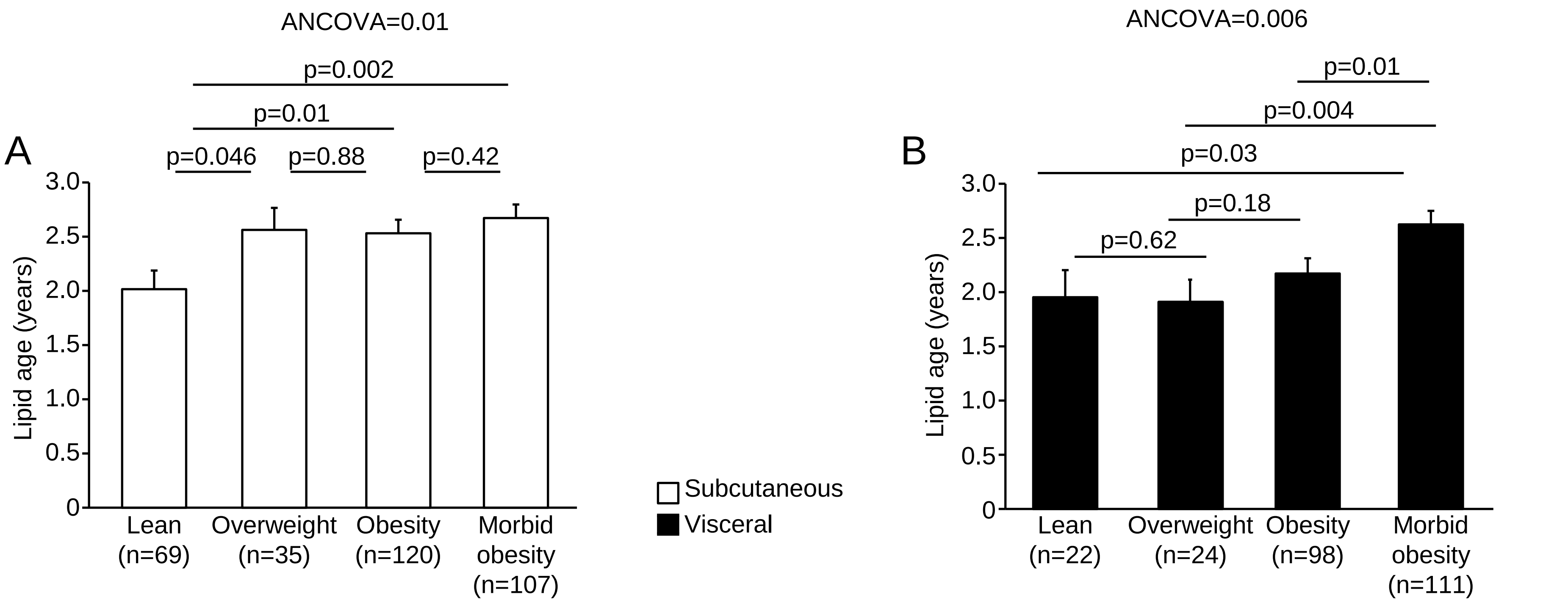

Supplementary Figure 1. ^{14}C lipid age in subcutaneous (A) and visceral (B) adipose tissue for four different BMI groups. Values are mean ± SEM and compared by analysis of co-variance (ANCOVA) and post-hoc test. n = number of subjects.

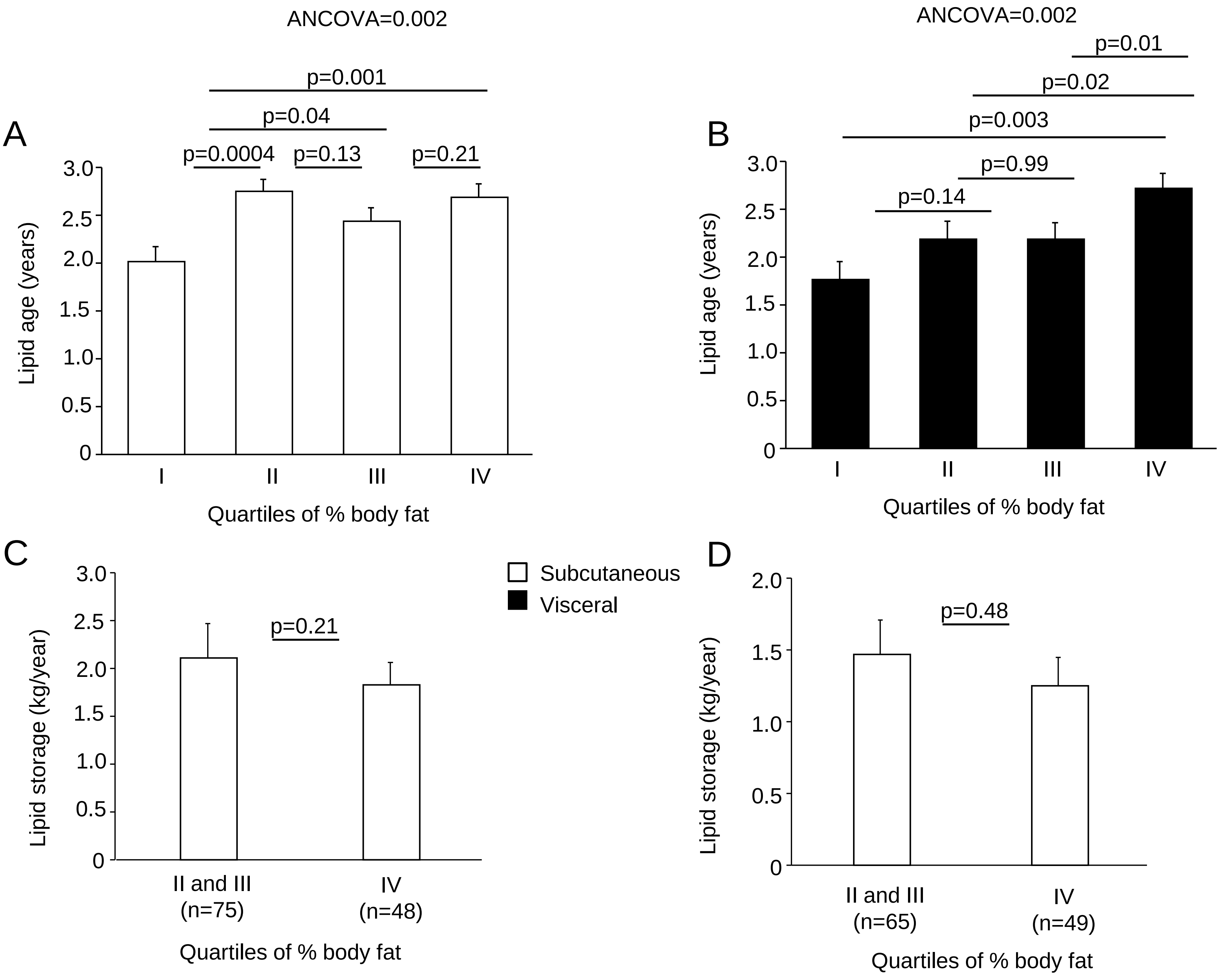

Supplementary Figure 2. ^{14}C lipid age and lipid storage in subcutaneous (A,C) and visceral (B,D) adipose tissue of subjects divided into quantiles of percentage total body fat. Values are mean ± SEM and compared by analysis of covariance (ANOVA) and post-host test. n = number of subjects

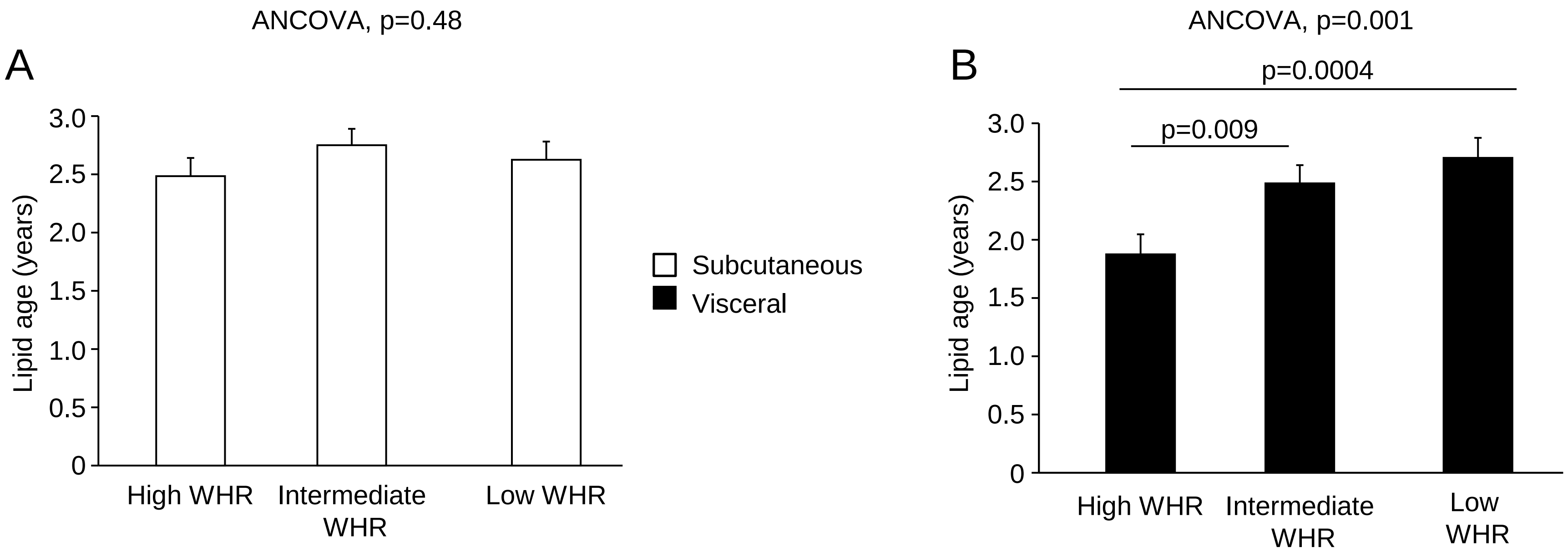

Supplementary Figure 3. ^{14}C lipid age in subjects with different waist-to-hip ratio (WHR). The subjects (n=210) were divided into tertiles of WHR. Data are shown for subcutaneous (A) and visceral (B) adipose tissue. Values are mean ± SEM and are compared by analysis of covariance (ANOVA) and post-host test.

Supplementary Table 1

Sample ID	Sample type [1]	DOB [2]	DOC [3]	Sex	BMI	F14C	F14C error, 2s	Delta 14C	Delta14C error, 2s	Lipid age (years)
ND169	Lipid SC	194703	201005	Male	24.4	1.0564	0.0068	48.7	6.8	2.0
ND169	Blood	194703	201005	Male	24.4	1.0493	0.0065	41.7	6.5	0.0
ND174	Lipid SC	193903	201008	Female	21.2	1.0550	0.0067	47.3	6.7	1.9
ND174	Blood	193903	201008	Female	21.2	1.0499	0.0064	42.2	6.4	0.4
ND175	Lipid SC	198405	201008	Male	25.8	1.0514	0.0067	43.8	6.7	0.9
ND175	Blood	198405	201008	Male	25.8	1.0495	0.0066	41.8	6.6	0.3
ND197	Lipid SC	195807	201011	Female	20.5	1.0575	0.0067	49.7	6.7	2.6
ND197	Blood	195807	201011	Female	20.5	1.0535	0.0069	45.8	6.9	1.7
ND211	Lipid SC	195804	201103	Female	22.1	1.0590	0.0070	51.2	7.0	3.2
ND211	Blood	195804	201103	Female	22.1	1.0504	0.0067	42.7	6.7	1.1
ND204	Lipid SC	194811	201101	Male	22.8	1.0528	0.0066	45.1	6.6	1.6
ND204	Blood	194811	201101	Male	22.8	1.0477	0.0066	40.0	6.6	0.0
ND164	Lipid SC	195503	201004	Female	34.9	1.0643	0.0069	56.6	6.9	3.6
ND164	Blood	195503	201004	Female	34.9	1.0537	0.0068	46.0	6.8	1.2
ND216	Lipid SC	193801	201104	Male	35.0	1.0564	0.0067	48.6	6.7	2.7
ND216	Blood	193801	201104	Male	35.0	1.0452	0.0066	37.5	6.6	0.0
ND180	Lipid SC	197106	201009	Female	44.1	1.0577	0.0067	49.9	6.7	2.6
ND180	Blood	197106	201009	Female	44.1	1.0501	0.0071	42.4	7.1	0.5
ND181	Lipid SC	196508	201009	Male	30.5	1.0538	0.0068	46.1	6.8	1.6
ND181	Blood	196508	201009	Male	30.5	1.0604	0.0109	52.6	10.9	3.2
ND228	Lipid SC	198401	201108	Male	31.4	1.0676	0.0438	59.7	43.8	5.3
ND228	Blood	198401	201108	Male	31.4	1.0433	0.0066	35.6	6.6	0.0
ND228	Dried Blood	198401	201108	Male	31.4	1.0456	0.0066	37.9	6.6	0.2
ND236	Lipid SC	198311	201111	Female	41.0	1.0551	0.0067	47.2	6.7	2.9
ND236	Blood	198311	201111	Female	41.0	1.0470	0.0067	39.2	6.7	0.9
ND236	Dried Blood	198311	201111	Female	41.0	1.0470	0.0066	39.2	6.6	0.9
ND238	Lipid SC	197405	201112	Male	23.5	1.0542	0.0069	46.4	6.9	2.8
ND238	Blood	197405	201112	Male	23.5	1.0518	0.0067	44.0	6.7	2.3
ND238	Dried Blood	197405	201112	Male	23.5	1.0443	0.0065	36.5	6.5	0.4
ND239	Lipid SC	194301	201201	Female	17.8	1.0639	0.0118	56.0	11.8	4.9
ND239	Blood	194301	201201	Female	17.8	1.0446	0.0065	36.8	6.5	0.5
ND239	Dried Blood	194301	201201	Female	17.8	1.0462	0.0067	38.4	6.7	0.9
ND246	Lipid SC	196306	201203	Male	31.6	1.0637	0.0074	55.7	7.4	5.0
ND246	Blood	196306	201203	Male	31.6	1.0491	0.0076	41.2	7.6	1.8
ND248	Lipid SC	195411	201203	Male	21.7	1.0438	0.0074	36.0	7.4	0.6
ND248	Blood	195411	201203	Male	21.7	1.0514	0.0077	43.5	7.7	2.4
ND249	Lipid SC	198012	201203	Male	21.9	1.0509	0.0074	43.0	7.4	2.3
ND249	Blood	198012	201203	Male	21.9	1.0484	0.0075	40.5	7.5	1.7
ND250	Lipid SC	195709	201203	Female	39.8	1.0581	0.0075	50.2	7.5	3.9
ND250	Blood	195709	201203	Female	39.8	1.0415	0.0076	33.7	7.6	0.2
ND251	Lipid SC	197211	201203	Male	33.7	1.0484	0.0074	40.6	7.4	1.7
ND251	Blood	197211	201203	Male	33.7	1.0507	0.0076	42.9	7.6	2.3
ND253	Lipid SC	197810	201205	Female	19.2	1.0471	0.0075	39.3	7.5	1.6
ND253	Blood	197810	201205	Female	19.2	1.0394	0.0072	31.6	7.2	0.1
ND255	Lipid SC	196107	201206	Female	21.8	1.0451	0.0224	37.2	22.4	1.2
ND255	Blood	196107	201206	Female	21.8	1.0442	0.0072	36.3	7.2	1.0
ND257	Lipid SC	198704	201206	Male	22.2	1.0469	0.0072	39.0	7.2	1.6
ND257	Blood	198704	201206	Male	22.2	1.0429	0.0073	35.0	7.3	0.7

1. Sample type (lipid subcutaneous, whole frozen blood, whole dried blood)
2. Patient date of birth (DOB)
3. Sample date of collection (DOC)

Supplementary Table 2. Characteristics of healthy and unhealthy obese classified according to ATP III criteria

Phenotype	Healthy (n=93)	Unhealthy (n=88)	p-value
Body mass index, kg/m^2	41 ± 5	42 ± 5	0.35
Abdominal subcutaneous fat mass, kg	3.6 ± 1.2	3.5 ± 0.8	0.51
Abdominal subcutaneous fat cell volume, picolitres	874 ± 173	968 ± 186	0.0004
Abdominal subcutaneous fat cell number X 10^7	456 ± 20	379 ± 12	0.0007

Values are Mean ± SD and compared by unpaired t-test. ATP III score 0=2 = healthy and 3-5 = unhealthy.

Supplementary Table 3. Clinical characteristics

Gender (males/females)	58/288
Age, years	42 (18-80)
BMI, kg/m^2	35 (18-60)
Waist-to-hip, ratio<br>Men (n=32)<br>Women (n=179)	 1.06 (0.97–1.12)<br>0.97 (0.76-1.29)
ANTIDIABETIC TREATMENT<br>Metformin /insulin/sulphonylurea/only diet	5/0/2/0
LIPID LOWERING DRUGS<br>Statins/other	9/0
ADIPOSE SAMPLES *<br>Paired/only subcutaneous/only visceral	 241/90/14

Values are mean and (range) for continuous variables. * Samples from one woman were lost during preparation.

Supplementary Table 4. Information on deceased subjects

Case no	*Age,years*	*Sex*	*BMI, kg/m²*	*Cause of death, disease, drugs*
1	33	Female	19.2	Traffic accident, internal injuries, drugs in the car and needles, amphetamine, opiates, methadone
2	50	Female	21.8	Suicide by drowning and drug poisoning, depression
3	30	Male	25.1	Chronic obstructive lung disease, renal failure, liver disease, cholecystitis, sepsis
4	25	Male	22.2	Accident, intoxication, methadone, mixed drug abuse
5	43	Male	31.0	Opiate intoxication, drug addict
6	52	Male	22.5	Suicide, depression, alcohol problem and earlier drug addict
7	65	Female	17.6	Suicide (hanging), depression
8	19	Male	20.8	Known drug addict, pregabalin, clonazepam
9	27	Male	26.0	Suicide (hanging), depression
10	25	Female	23.3	Car accident, multipole internal injuries
11	48	Male	31.1	Accident, unintentional falling injuries and alcohol poisoning
12	60	Male	27.0	Diabetic coma, alcohol addict, Type 2 diabetes, hypertension, metformin
13	54	Male	31.6	Acute ischemic cardiac muscle damage, was falling when cycling, natural death
14	71	Male	21.0	Coronary heart/heart disease, retirement home, dementia
15	27	Male	22.6	Drug addict, overdose, hashish, methadone, amphetamine, pneumonia
16	79	Male	30.8	Cramp, heart failure, cardiomegaly, natural death
17	62	Male	29.4	Cerebral haemorrhage, heart disease, hypertension
18	23	Female	18.9	Suicide (hanging)
19	65	Male	24.8	Natural death
20	62	Male	33.1	Blood clot in the lung
21	51	Male	25.2	Suicide (depression)
22	24	Male	30.8	Alcohol accident, intoxication
23	58	Male	24.8	Suicide (hanging)
24	29	Male	25.9	Intoxication (no suicide) - depressed
25	53	Male	28.4	Suicide, intoxication, probably by CO2
26	20	Male	23.3	Suicide (hanging)
27	47	Male	31.7	Suicide (hanging), alcohol abuser

The time between found dead and adipose biopsy during autopsy varied between 1-3 days
